# Systemic lupus erythematosus manifestation following COVID-19: a case report

**DOI:** 10.1186/s13256-020-02582-8

**Published:** 2021-01-25

**Authors:** Batool Zamani, Seyed-Masoud Moeini Taba, Mohammad Shayestehpour

**Affiliations:** 1 Autoimmune Diseases Research Center, Kashan University of Medical Sciences, Kashan, Iran; 2 Department of Microbiology and Immunology, Faculty of Medicine, Kashan University of Medical Sciences, Kashan, Iran

**Keywords:** Autoantibodies, Autoimmune diseases, COVID-19, Systemic lupus erythematosus

## Abstract

**Background:**

Systemic lupus erythematosus (SLE) is a complex and challenging autoimmune disease. Severe acute respiratory syndrome coronavirus 2 (SARS‑CoV‑2) is a novel viral agent that can cause a life-threatening respiratory disorder named coronavirus disease 2019 (COVID‑19). Association between SARS‑CoV‑2 and SLE is not clear. We reported the first case of SLE manifestation following COVID-19.

**Case presentation:**

A 39-year-old Iranian/Persian man with complaints of fever, scaling on the palms of the hands and feet, lower extremity edema, and ankle swelling was referred to Kashan Rheumatology Clinic in 2020. He was infected with SARS-CoV-2 2 months ago. The patient had proteinuria and was positive for SLE laboratory tests. After one week of treatment with prednisolone (30 mg daily) and hydroxychloroquine, paresthesia, proteinuria, and edema continued. The patient was treated with pulse methylprednisolone (1000 mg for three consecutive days), gabapentin, and vitamin B (300 mg daily), which reduced paresthesia.

**Conclusions:**

This is the first case of SLE manifestation following COVID-19. SARS-CoV-2 may produce autoantibodies or develop the clinical features of subclinical SLE.

## Introduction

Systemic lupus erythematosus (SLE) is an autoimmune disease characterized by the breaking of tolerance to nuclear self-antigens and the production of pathogenic autoantibodies. This complex and challenging disease can involve several organs in the body such as skin, eyes, kidney, heart, muscles, and the joints [1]. Previous studies reported that some viruses may be implicated in the etiology of SLE. Epstein–Barr virus (EBV) is as potential causal agent of SLE. In addition, cytomegalovirus, parvovirus B19, and retroviruses can be possible triggers for SLE. Immune reactions against viral antigens may turn against the self-antigens that can lead to autoimmunity (molecular mimicry) [2]. There are reports of several cases of SLE that have manifested following acute viral infections (Dengue fever virus) [3].

Severe acute respiratory syndrome coronavirus 2 (SARS‑CoV‑2) is a novel viral agent that has caused coronavirus disease 2019 (COVID‑19). This acute viral infection is frequently associated with respiratory failure, pneumonia, acute respiratory distress syndrome (ARDS), and sepsis [4]. A fulminant increase in the serum pro-inflammatory cytokines including, IL-1, IL-6, IL-12, IFN-γ, TGFβ, and chemokines such as CCL2, CXCL9, CXCL10 was detected in severe cases of COVID‑19 [5]. Although a few case reports have been published on the possible association between coronavirus and autoimmune disorders, the role of this virus in autoimmunity is not clear [6]. To date, several cases have been reported patients with lupus who have been infected with COVID-19 [7], but no data have been published on the onset of clinical manifestations of SLE after getting COVID-19. We reported the first case of SLE manifesting following COVID-19.

## Case presentation

A 39-year-old Iranian/Persian man with complaints of fever (38 °C), scaling on the palms of the hands and feet, lower extremity edema, and ankle swelling was referred to Kashan Rheumatology Clinic in 2020.

2 months ago, the patient was referred with complaints of fever, dry cough, shortness of breath, and wheezing. Upon arrival he had a high temperature (38 ℃), a respiratory rate of 22 breaths per minute, a heart rate of 110/min, a blood pressure of 100/70 and oxygen saturation of 93%. The patient had no history of alcohol consumption and cigarette smoking. The result of laboratory tests showed leukopenia (white blood cell (WBC): 4200/mm^3^), thrombocytopenia (platelet count: 73,000/mm^3^), high C-reactive protein (CRP: 43 mg/L), hemoglobinemia (hemoglobin level: 11.2 g/dL), and normal liver function tests.

**Fig. 1:**
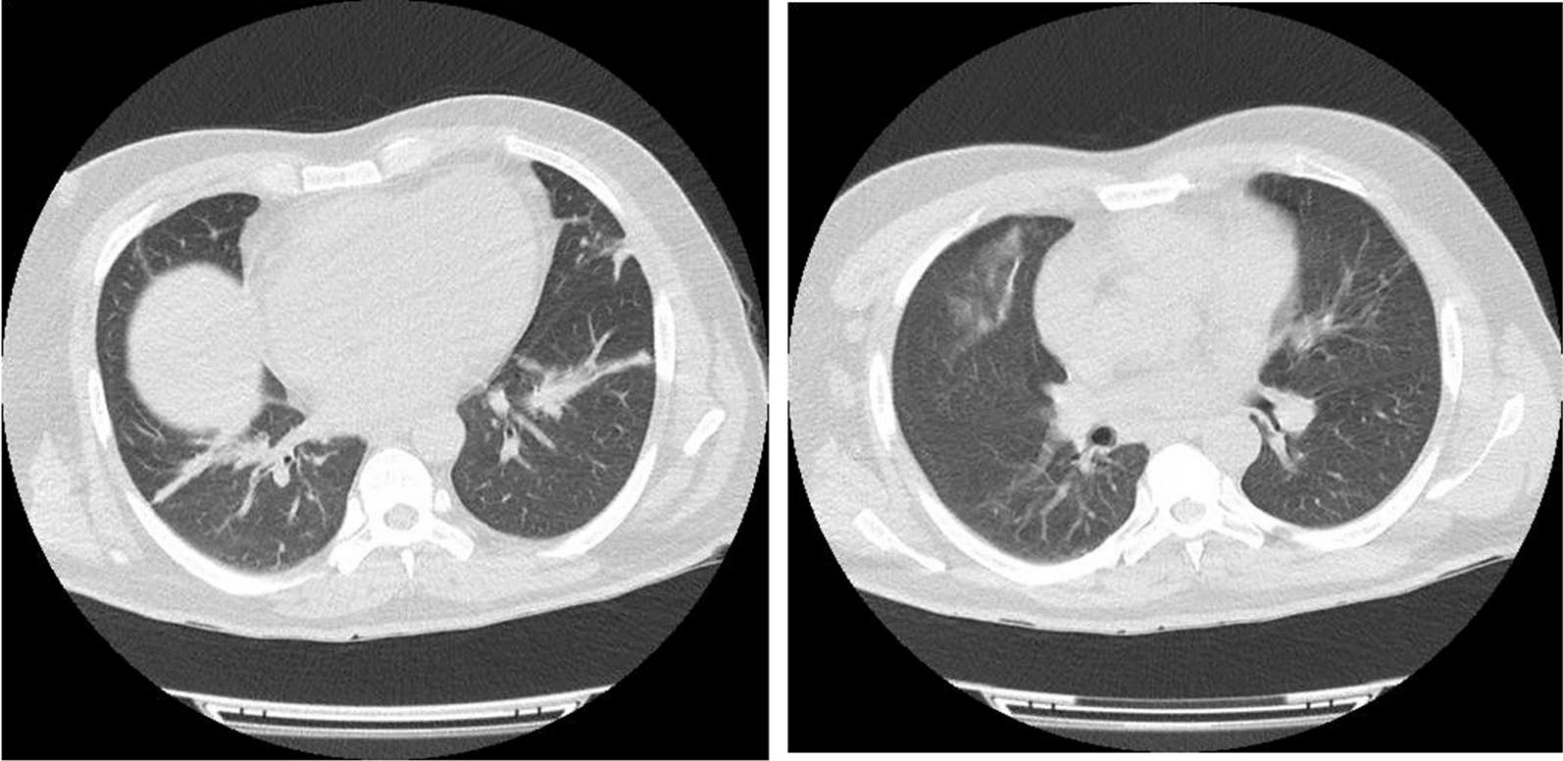
Chest computed tomography images of the patient with COVID-19. Ground-glass opacity nodules are seen in the lower lobes of both lungs

Computed tomography (CT) of the chest showed two ground-glass opacity nodules in the lower lobes of both lungs (Fig. 1). SARS-CoV-2 was detected in the nasal swab by reverse-transcription polymerase chain reaction test (RT-PCR). The patient did not need intensive care unit (ICU) admission and underwent outpatient treatment for COVID-19. He was treated with 400 mg oral dose of hydroxychloroquine twice on the first day and 200 mg twice daily for an additional 6 days.

The patient recovered after 4 weeks, but gradually, urticaria-like skin lesions and erythematous rashes appeared on the chest, upper and lower limbs with itching. The patient developed scaling on the palms of the hands and feet and hyperkeratosis of the soles of the feet. Lower extremity edema and ankle swelling were also added to the complications of the disease. The patient suffered from a weight loss of about 15 kg, anorexia, and headache during 2 months. He was suffering from hyperesthesia of lower extremity on both sides when referred to the rheumatology clinic. He felt severe burning and pain when his feet were touched. The tendon reflexes and strength of the lower and upper limbs were normal. The results of primary laboratory tests were as follows: platelet count, 73,000/mm^3^ (150,000–400,000/mm^3^); white blood cell count, 4200/mm^3^ (4500–11,000/mm^3^); (14–18 g/dL); C-reactive protein (CRP) level, 34 mg/L (< 10 mg/L); erythrocyte sedimentation rate (ESR), 74 mm/hour (0–20 mm/hour); lactate dehydrogenase (LDH), 437 U/L (150–450 U/L); troponin I, 3 μg/L (< 0.03 μg/L). Electrolytes, kidney and liver function tests were normal.

Due to the observation of bicytopenia, the patient's peripheral blood smear (PBS) was evaluated by a hematologist. Toxic granulation was observed in the PBS while blast cells and schistocytes were not seen. Patient had a normal echocardiogram. SARS-CoV-2 genome was not detected by RT-PCR. IgG antibodies against SARS-CoV-2 were detectable by the enzyme-linked immunosorbent assay (ELISA) (Pishtaz Teb Zaman, Tehran, Iran), but IgM antibodies against SARS-CoV-2 were negative (Pishtaz Teb Zaman, Tehran, Iran) in the serum sample. Urine analysis showed 2+ proteinuria and 550 mg of protein was measured in the 24-h urine sample. Electromyography (EMG) and nerve conduction velocity (NCV) showed motor and sensory polyneuropathies.

**Fig. 2:**
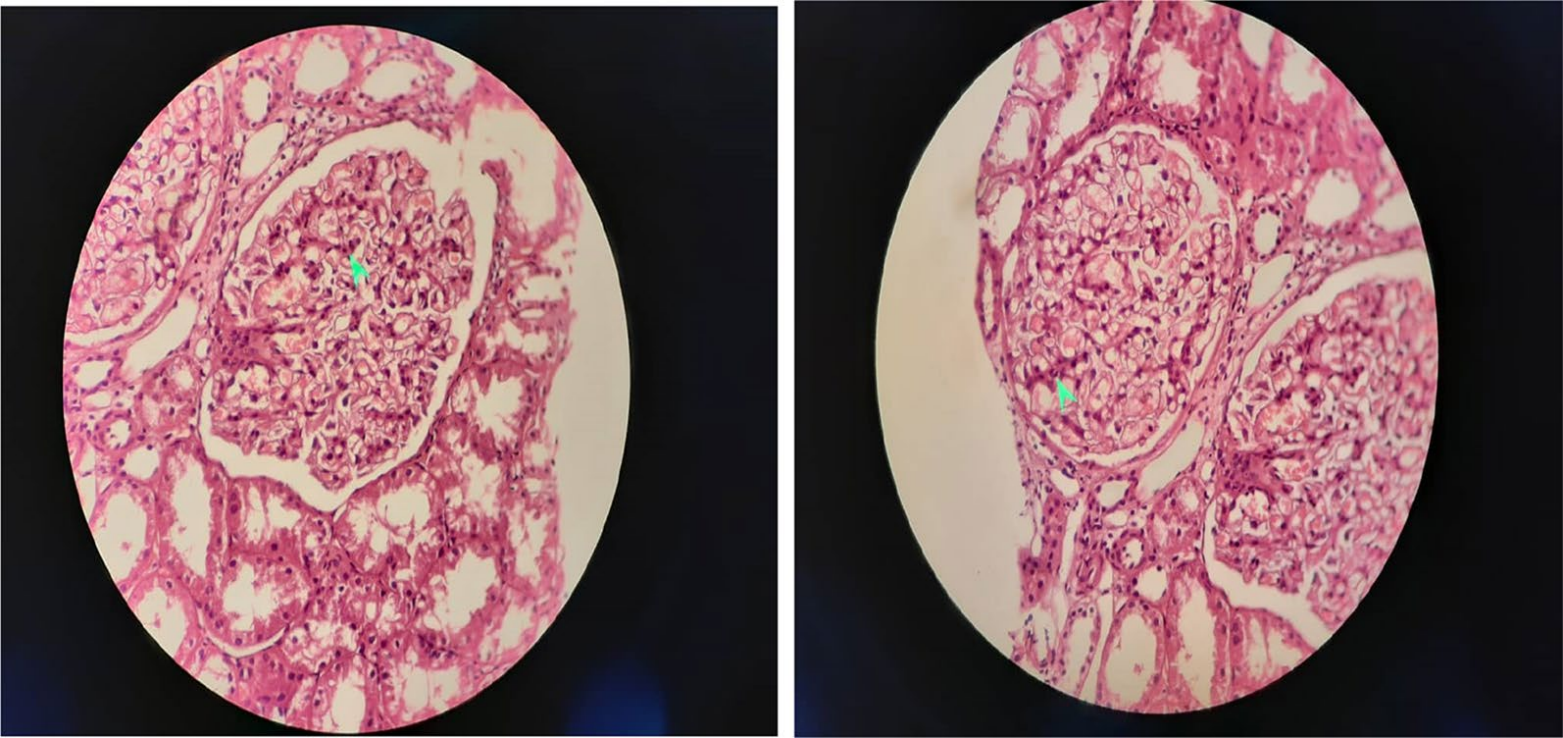
The histopathology of kidney biopsy in the patient with SLE and COVID-19. Arrow shows inflammatory cell infiltration. A mild mesangial hypercellularity and mild fibrosis is seen in the background

The patient was suspected of having SLE. The results of laboratory tests to diagnosis systemic lupus erythematosus are as follows: total complement activity (CH50), 45 (50–150); complement C3 protein, 133 mg/dL (90–180 mg/dL); complement C4 protein, 14 mg/dL (10–40 mg/dL); anti-La/SSB antibodies, 160 U/ml (< 12 U/mL); anti-SSA/Ro, 200 U/mL (< 25 U/mL); anti-cyclic citrullinated peptides (anti-CCP) antibodies, 48 IU/mL (< 20 IU/mL) ; anti-double-stranded deoxyribonucleic acid antibody (anti-dsDNA), 70 IU/mL (< 35 IU/mL); fluorescence antinuclear antibody (FANA), 1/160. Anticardiolipin, lupus anticoagulant, anti-beta-2 glycoprotein 1, and anti-neutrophil cytoplasmic antibodies (C-ANCA, P-ANCA) were negative. The patient’s kidney biopsy showed a mild mesangial hypercellularity (lupus nephritis class I). Mild intermediate fibrosis was observed in trichrome staining of tissue (Fig. 2).

The treatment with pulse methylprednisolone (1000 mg for three consecutive days) was started and continued with hydroxychloroquine and prednisolone (30 mg daily). Platelets were reduced to 100,000/mm^3^ and hemoglobin to 11 g/dL, but paresthesia, proteinuria, and edema continued. The patient has received monthly pulse doses of 1000-mg IV cyclophosphamide. The patient was discharged while being given hydroxychloroquine, prednisolone (10 mg daily), cyclophosphamide, gabapentin, and vitamin B (300 mg daily).

The patient was followed up after the 6-month. Paresthesia was improved. The laboratory tests (CBC, ESR, CRP, T3, T4) were normal and urine protein was 230 mg/daily. Anti-double-stranded DNA antibody reduced to the normal range (< 35 IU/mL).

## Discussion

We presented a case of systemic lupus erythematosus associated with COVID-19 in a 39-year-old man. The patient developed COVID-19, and after about 2 months presented subsequently with manifestations of SLE. Recent studies have shown some clinical situations caused by chronic viral infections in patients with SLE. The researchers reported cases of SLE reactivated in human immunodeficiency virus (HIV) and hepatitis C virus (HCV) patients after antiviral therapy. In addition, acute viral infections such as parvovirus B19 and EBV can mimic lupus, trigger lupus, or trigger SLE flares [2].

SARS-CoV-2 is a positive-sense single-stranded ribonucleic acid (RNA) virus that causes coronavirus disease 2019 (COVID-19). This acute viral infection is associated with a life-threatening respiratory disease [4]. We found a patient who showed clinical signs of lupus 2 months after getting COVID-19. Rajadhyaksha *et al.* reported a case of Dengue fever virus evolving into SLE and lupus nephritis. They found that dengue virus has triggered a dysfunctional immune response and resulted in the developing of autoimmunity, SLE, and lupus nephritis [3]. There are no published data on association of coronaviruses with SLE, but several studies have linked coronaviruses with another autoimmune disease such as multiple sclerosis (MS) and rheumatoid arthritis (RA). Joo *et al.* were observed an association between exposure to respiratory viral infections (including coronavirus, parainfluenza, metapneumovirus) for 6–7 weeks and the increase in incident RA. They concluded that coronaviruses may have the capacity to trigger RA [6]. A severe immune activation in response to SARS-CoV-2 infection has seen in some patients infected with COVID-19 that resulted in acute respiratory distress syndrome, and a cytokine storm. SARS-CoV-2 increases interferon gamma, tumor necrosis factor-α, macrophage inflammatory protein-1 alpha, IL-2, IL-6, IL-7, IL-10, in patients, that show a form of secondary hemophagocytic lymphohistiocytosis or macrophage activation syndrome (sHLH/MAS). Previous studies reported HLH in the background of SLE [8]. We found a case of SLE related to the new coronavirus (SARS‑CoV‑2). Acute infection with coronavirus may produce autoantibodies, such as anti-CCP antibodies and antinuclear antibodies [9].

## Conclusion

Although single-case observations have limitations, the presented case report shows the possible role of novel coronavirus in the manifestation of SLE. Future reports can support or refute this hypothesis. We need to wait and see for more cases of autoimmune disorders to declare themselves following viral infections like COVID-19.

## Data Availability

No additional file is available for this study; all the data are included in the manuscript.

## Abbreviations

CT: Computed tomography
COVID‑19: Coronavirus disease 2019
SARS‑CoV‑2: Severe acute respiratory syndrome coronavirus
SLE: Systemic lupus erythematosus
